# Structural insight to mutation effects uncover a common allosteric site in class C GPCRs

**DOI:** 10.1093/bioinformatics/btw784

**Published:** 2016-12-22

**Authors:** Kasper Harpsøe, Michael W Boesgaard, Christian Munk, Hans Bräuner-Osborne, David E Gloriam

**Affiliations:** Department of Drug Design and Pharmacology, Faculty of Health and Medical Sciences, University of Copenhagen, Copenhagen, Denmark

## Abstract

**Motivation:**

Class C G protein-coupled receptors (GPCRs) regulate important physiological functions and allosteric modulators binding to the transmembrane domain constitute an attractive and, due to a lack of structural insight, a virtually unexplored potential for therapeutics and the food industry. Combining pharmacological site-directed mutagenesis data with the recent class C GPCR experimental structures will provide a foundation for rational design of new therapeutics.

**Results:**

We uncover one common site for both positive and negative modulators with different amino acid layouts that can be utilized to obtain selectivity. Additionally, we show a large potential for structure-based modulator design, especially for four orphan receptors with high similarity to the crystal structures.

**Availability and Implementation:**

All collated mutagenesis data is available in the GPCRdb mutation browser at http://gpcrdb.org/mutations/ and can be analyzed online or downloaded in excel format.

**Supplementary information:**

Supplementary data are available at *Bioinformatics* online.

## 1 Introduction

Class C of G protein-coupled receptors (GPCRs) consists of five receptor families (Alexander *et al.*, 2015): eight metabotropic glutamate receptors (mGlu_1-8_) sub-divided into three groups (Conn and Pin, 1997), two GABA receptors (GABA_B1-2_) (Pinard *et al.*, 2010), three taste 1 receptors, (TAS1R1-3) (Treesukosol *et al.*, 2011), the calcium-sensing (CaS) and GPRC_6_ receptors, and seven orphan receptors, GPR156, GPR158, GPR179 and GPRC5A-D. The mGlu and GABA_B_ receptors regulate neuronal transmission throughout the central nervous system and have been implicated in many high-interest diseases as e.g. Parkinson's. In addition to the well-known functions of the CaS receptor it has been implicated in e.g. Alzheimer's disease. The taste 1 receptors are responsible for umami and sweet taste and a broad expression pattern may indicate other physiological functions. Physiological function and therapeutic utilization of orphan receptors are unknown and are being debated for the GPRC_6_ receptor (Clemmensen *et al.*, 2014) (Supplementary Table S1).

Allosteric modulators (AMs) that bind in the transmembrane domain (TMD) comprised of seven helices (TM1-7) have now been discovered for all non-orphan class C GPCR families with potential therapeutic advantages by enhancing or attenuating the normal receptor response without interfering with the N-terminal domain orthosteric site (Lu *et al.*, 2014). Furthermore, AMs potentially offer better receptor subtype selectivity and biased signalling, which in concert can lead to fewer undesirable side-effects (Nickols and Conn, 2014). This allosteric site in class C GPCRs constitute targets for therapeutics as well as for the food industry (Supplementary Table S1), exemplified by the marketed drug cinacalcet, a CaS receptor positive AM (PAM) (Nemeth and Shoback, 2013) and a Parkinson's disease clinical trial with dipraglurant, a mGlu_5_ negative AM (NAM) by Addex Pharma (Tison *et al.*, 2016).

The binding sites of class C GPCR AMs have been extensively studied using site-directed mutagenesis and receptor structure models (Gregory and Conn, 2015), but the lack of class C structures limited the resolutions of these analyses, hampering the translation of mutagenesis effects and models into pharmacological mechanisms and prospective rational structure-based drug design. Similarly to previously published work on class A GPCRs (Hulme, 2013), the recent mGlu structures (Christopher *et al.*, 2015; Dore *et al.*, 2014; Wu *et al.*, 2014) has herein opened up for the first consistent comparison of all published class C GPCR mutants in the TMD. We pinpoint the location of the allosteric sites, and integrate mutant and structural data towards rational design of new modulators.

## 2 Methods

### 2.1 Annotation of literature single-point mutations

We manually annotated 1670 data points from 35 publications with single-point mutations tested on 70 AMs and made them available online in the GPCR database, GPCRdb (Isberg *et al.*, 2016). These mutants cover 99 TMD helix and several extracellular loop positions within the CaS, GABA_B2_ (not shown, as all reported single-point mutations had low effect on PAM function), GPRC_6_, mGlu_1_, mGlu_2_, mGlu_4_, mGlu_5_ and TAS1R3 receptors. Residue positions, numbered according to the GPCRdb generic scheme (Isberg *et al.*, 2015), were considered as potential ligand binding if they have >5-fold effect on AM binding or function (Figs 2A, 3A).

### 2.2 Sequence alignment, similarities and phylogenetic trees

**Fig. 1:**
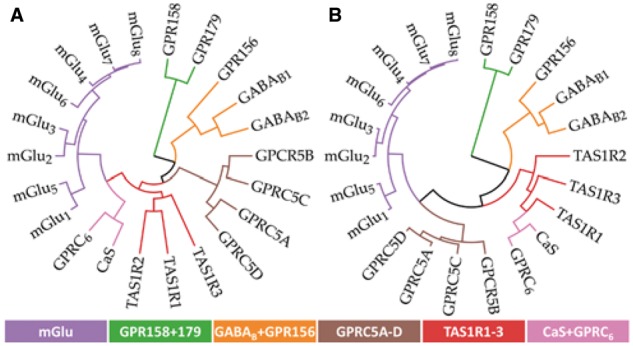
Phylogenetic trees of class C GPCRs based on (**A**) the TMD and (**B**) the common allosteric site. Both trees group according to the families based on endogenous ligands binding in the N-terminus (Color version of this figure is available at *Bioinformatics* online.)

The sequence alignments and similarities (Supplementary Figs S1, S2 and Table S2) of the 22 human class C GPCRs was calculated and downloaded from GPCRdb (Isberg *et al.*, 2016) The phylogenetic trees (Fig. 1) were calculated with the PHYLIP package, v. 3.695 (Felsenstein, 1989), based on the above alignments using the *protdist* and *neighbor* programs with UPGMA clustering and rendered by an in-house javascript using D3.js.

### 2.3 Crystal structure binding site residues

**Fig. 2:**
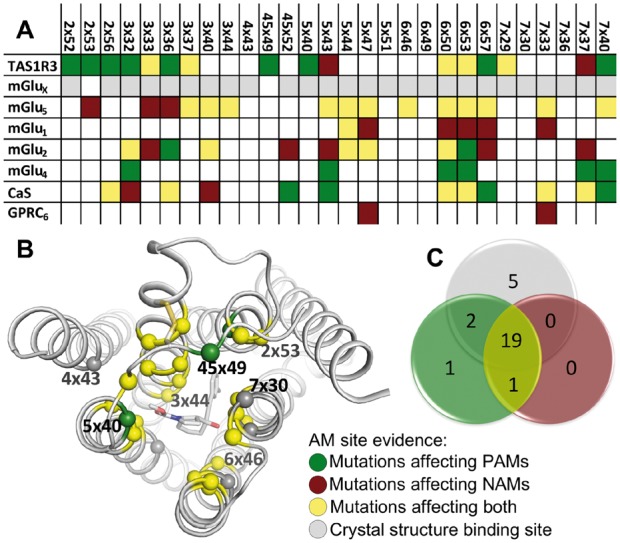
Common allosteric binding site. (**A**) 28 binding site positions from crystal structures and mutagenesis data. (**B**) Mapping onto the mGlu_5_ structure (Cα-spheres) including the NAM, mavoglurant. (**C**) Venn diagram (Color version of this figure is available at *Bioinformatics* online.)

**Fig. 3:**
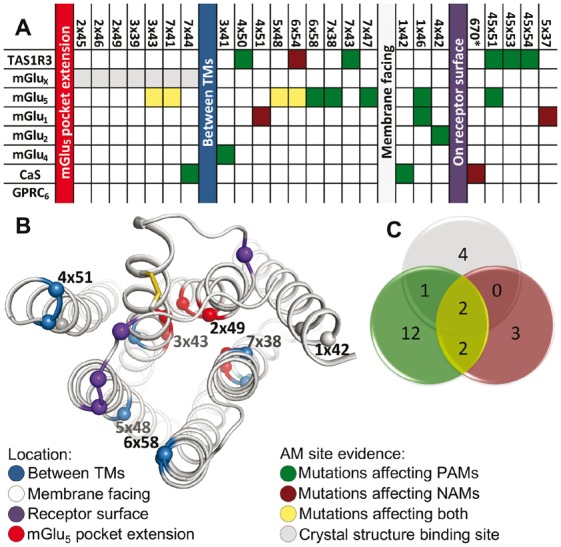
Positions outside the common allosteric binding site. (**A**) 24 positions from crystal structures and mutagenesis data. (**B**) Mapping onto the mGlu_5_ structure (Cα-spheres; 45x54 is absent in the structure) (**C**) Venn diagram (Color version of this figure is available at *Bioinformatics* online.)

The mGlu_5_ (PDB codes, 4OO9, 5CGC and 5CGD) and mGlu_1_ (PDB code 4OR2) crystal structures were downloaded from the Protein Data Bank (www.rcsb.org) and the PyMOL Molecular Graphics System, version 1.8, Schrödinger, LLC was used to prepare Figures 2B and 3B and determine the residue positions in the allosteric binding site by selecting residues with Cα or side chain atoms ≤5 Å of any co-crystallized NAM atom.

## 3 Results

### 3.1 The class C GPCR families group consistently for the transmembrane domain and allosteric modulator site

Class C GPCRs are classified into families by endogenous ligand (Southan *et al.*, 2016) (Supplementary Table S1), which bind in the extracellular N-terminal domain, while the reported AMs bind in the distinct TMD. Thus, it is crucial to establish if these receptor families are valid also for the analysis of AMs. We constructed phylogenetic trees based on the TMD and common allosteric site (below) sequence alignments (Figs 1, S1 and S2). In both trees, the receptor families with a known physiological ligand display a coherent grouping of all members. In addition, the orphan receptors are divided into two groups with the exception of GPR156, which, in agreement with earlier reports (Calver *et al.*, 2003), groups with the GABA_B_ receptors. This confirms that the established receptor families can indeed serve as a basis to map allosteric data, and as target profiles for ligand selectivity and inference.

### 3.2 Class C GPCR negative and positive allosteric modulators bind in one common transmembrane domain site

An overall TMD allosteric site in the class C GPCRs can be delineated from the mGlu structures and the collated mutagenesis data. In total, 28 residue positions line the TMD pocket and together represent the overall potential AM contacts (Fig. 2). Notably, these exhibit strong experimental evidence: 26 positions are in close vicinity (≤5 Å) of the co-crystallized modulators, and 23 have demonstrated effect (>5-fold) upon mutagenesis (Fig. 2A). Nine AM binding hotspots, 2x56, 3x32, 3x36, 5x43, 6x50, 6x53, 6x57, 7x37 and 7x40, span three receptor families. The latter six positions are supported by mutant effect on 10 or more different ligands while mutations in the central part of the binding site, i.e. positions 6x50 and 6x53 show effect on 37 and 24 different ligands, respectively, in six different receptors (Supplementary Table S3). Furthermore, 68% of the positions re-occur in at least two families demonstrating a substantial overlap of the class C GPCR modulator sites. Structural mapping pinpoints an allosteric site that, as for most class A GPCR ligands, is located mainly between TM3 and TM5-7, with additional contacts to TM2 and the second extracellular loop (Fig. 2B). Notably, 87% of the mutated binding site positions are involved in both NAM and PAM binding and/or function (Fig. 2A, C) and the concordant structural and mutagenesis data strongly suggests the existence of a common allosteric binding site for both types of modulators with substantial overlap across the class C receptor families.

### 3.3 The second extracellular loop is involved in binding

The second extracellular loop of GPCRs typically forms a disulphide bridge to the top of TM3, placing the loop as a lid on the TMD pocket (Wu *et al.*, 2014), often in direct ligand contact (de Graaf *et al.*, 2008). It is constituted by a pair of cysteine residues (3x29 and 45x50) only absent in GPR156, GPRC5A and GPRC5D (Supplementary Fig. S1); indicating that the second extracellular loop is close to the AM site in nearly all class C GPCRs. Mutagenesis data reports effect on the position before (45x49) and four consecutive positions after (45x51-54) the conserved cysteine (Figs 2A, B, 3A, B and online in GPCRdb), while the mGlu structures only show one position (45x52) in direct contact with a NAM and an additional position (45x49) with the side chain pointing towards the AM site. This is expected as the many class A GPCR structures (Wheatley *et al.*, 2012) show the flexibility and varying length of the second extracellular loop to bring alternative positions into vicinity of the AM site.

### 3.4 The mGlu_5_ receptor features a unique site extension

The mGlu_5_ receptor structures contain a unique site extension located between TM2, 3 and 7 (Fig. 3A, B) that can be exploited to achieve selective NAM binding (Harpsøe *et al.*, 2015). Its uniqueness is supported by mutagenesis of the site extension position 7x41 in four different receptors with a selective effect in mGlu_5_ (Supplementary Table S3 and online in GPCRdb), and a gateway position 3x40 holding a mGlu_5_-specific Pro, which significantly decreases or abolishes AM binding or activity when mutated (Gregory *et al.*, 2013; Mølck *et al.*, 2012). Most other class C GPCRs contain bulkier sidechains in position 3x40 and in another crucial position, Gly2x49 (Harpsøe *et al.*, 2015) (Supplementary Fig. S1). In spite of this, a 6.7-fold PAM affinity decrease by a CaS receptor mutation in position 7x44 (Fig. 3A) in one of two assays (Leach *et al.*, 2016) challenges the uniqueness of the site extension. However, as a drastic F3x40A mutation in the site extension gateway position does not support this finding, it is tempting to consider it as an experimental outlier. In all, the data suggest that the extension of the modulator site in mGlu_5_ is unique not only within the mGlu receptors, but within all class C GPCRs.

### 3.5 A TM1 mutation may reveal a group I mGlu receptor dimer interface PAM site

In contrast to the clearly overlapping data in the common allosteric site (Fig. 2) several positions outside the common allosteric site have shown only PAM or NAM effect (Fig. 3A). However, as the mutational effects can be rationalized by other reasons (see below) they are not considered as functionally specific sites.

One exception is position 1x46 that face the cell membrane, where a Phe-to-Ile mutation abolishes the effect of the mGlu_1_ and mGlu_5_ PAM, CPPHA (Chen *et al.*, 2008), while it has no effect on other PAMs and NAMs (online in GPCRdb). The mGlu_1_ structure depicts a dimeric complex implicating TM1 of both protomers (Wu *et al.*, 2014) and, though the functional dimer interface of mGlu_2_ has been shown to involve TM4-6 (Xue *et al.*, 2015), it may be functionally important in group I mGlu receptors. The interface in this inactive state structure does not directly involve Phe1x46, but it may do so in the active state conformation of the receptor and take part in forming a specific PAM site explaining the observed mutational effects for CPPHA. Regardless of TM1 being part of a functional dimer interface, the fact remains, that among 22 positions only mutation of 1x46 showed effect on CPPHA modulation and it does not compete with modulators binding in the common allosteric site (Gregory *et al.*, 2011) showing the presence of at least one additional allosteric site.

### 3.6 Three TM5 and 6 mutations hamper the interactions of other adjacent residues

The combination of structures and mutagenesis data also provides the opportunity to filter out the data points that are not related to a direct receptor-ligand interaction but rather reflects a secondary effect. Mutation of 5x48 can affect modulator binding in mGlu_5_ but not in mGlu_2_ and mGlu_4_ and the effect was only observed for two of 30 tested AMs (Supplementary Table S3). An explanation is provided from different mGlu_5_-NAM structure complexes, wherein Gly5x48 allows for the adjacent Trp6x50 to alternate between the NAM site (Christopher *et al.*, 2015) and packing between TM5-6 (Dore *et al.*, 2014). Apart from mGlu_5_, only mGlu_1_, GABA_B1-2_, TAS1R1 and GPR156 have a Gly in 5x48 potentially allowing the induced fit of modulators through Trp6x50. Furthermore, 6x58 and 6x54 are located outside of the binding site but have exhibited up to 10- and 66-fold effects, respectively (Gregory *et al.*, 2013). Structural analysis show these residues to have stabilizing interactions to adjacent positions that mediate AM contacts (5x44, 6x53 and 6x57), e.g. in mGlu_5_ Tyr6x57 display π-π interactions to Phe6x58 (Dore *et al.*, 2014).

### 3.7 Glycine mutations perturb TM4 and 7 helical structure

Relative to other residues, glycines allow for additional backbone torsional angles and introduce more flexibility (Bywater and Veryazov, 2015). Mutation of two Gly positions, 4x42 and 7x43, that face the cell membrane and TM6, respectively, have been shown to affect modulator binding. Specifically, a Gly-to-Val mutation of position 4x42 yielded >55-fold effect on the mGlu_2_ PAM, JNJ-41482012 (Farinha *et al.*, 2015), and a Gly-to-Ala/Val mutation in 7x43 reduced/abolished activation of TAS1R3 by the allosteric agonist NHDC (Winnig *et al.*, 2007). Importantly, mutation in 4x42 has no effect when the native residue is not a Gly: Val-to-Ile and Leu-to-Val in mGlu_1_ and mGlu_4_, respectively, have no effect on several AMs (online in GPCRdb). Together, this indicates that these subtype specific Gly residues are associated with distinct receptor structures, such as a helical kink or rotation implicating additional class C GPCRs with a Gly at 4x42 (GPR179) or 7x43 (CaS and GPRC_6_ receptors) (Supplementary Fig. S1). It would be of interest to mutate Gly4x42 in the CaS and GPRC_6_ receptors, whereas mutations of Gly7x43 can currently not be tested on the orphan GPR179 due to lack of ligands.

### 3.8 Mutated positions with weak and rare effects

The remaining mutated positions in Figure 3, 1x42, 3x41, 4x50, 4x51, 5x37, 7x38 and 7x47, are distant to the common allosteric site and have relatively subtle effects reported for only one AM on one receptor (Supplementary Table S3). Of note, the AMs affected by mutations in these positions are all more pronouncedly affected by mutation in other positions within the common allosteric site. Taken together, it is fair to conclude that these mutants probably give their effect through other processes, such as e.g. binding site entry/exit or stabilization of an (in)active receptor state, or represent experimental outliers.

## 4 Discussion

The available structural templates, which are all NAM-complexes, map as well to the PAM as the NAM mutagenesis data (Fig. 2). This is in line with the many class A GPCR-agonist and -antagonist structure complexes which exhibit moderate differences in the TMD ligand binding site (Kruse *et al.*, 2013). This suggests that the mGlu_1_ and mGlu_5_ structures could be valid templates also for the design of PAMs. However, to avoid bias toward NAM activity, it is advisable to first optimize the structural templates around a high-affinity PAM, which has previously been successful in GPCR virtual screening (Vilar *et al.*, 2011). Furthermore, AMs have increasingly been associated with biased agonism, including CaS (Cook *et al.*, 2015; Leach *et al.*, 2016) and mGlu_5_ (Sengmany and Gregory, 2016) receptors, and it could be expected that several already existing and future AMs share this phenomenon.

An advantage of allosteric ligands is their ability to achieve higher receptor target selectivity. As shown in Figure 2, the allosteric site is largely the same across the class C receptor families, which could imply less selective binding. However, sequence alignment of the 28 positions that constitute the common allosteric site shows that, in spite of many positions displaying more than 60% conservation of side chain properties (e.g. 86% of the receptors contain an aromatic residue in position 6x53—Supplementary Fig. S2), there are a number of family- and subtype-unique residues. Thus, as shown for the mGlu receptors (Harpsøe *et al.*, 2015), selective modulators may be achieved by exploiting these as selectivity hotspots. This is also supported by the known chemistry, as prototypical AMs (Supplementary Fig. S3) display highly diverse structures.

Modelling of class C GPCR-AM complexes has become more accessible with the possibility to utilize the mGlu structures in combination with our collation of literature mutations, which are available for visualization and download in GPCRdb. We previously demonstrated that such data was sufficient to build conclusive models explaining mGlu NAM selectivity (Harpsøe *et al.*, 2015). Interestingly, the receptors that displays the closest homology within the allosteric site (50–64%) are the orphan GPRC5A-D receptors (Supplementary Table S2). This is encouraging for the prospects of identifying ligands through receptor structure-based virtual screening. Notably, since the orphan receptors lack the extracellular domain (Kniazeff *et al.*, 2011), it is plausible that the TMD site constitutes their orthosteric site in line with the fact that other class C GPCRs can be activated via the allosteric site, even without the N-terminal domain (Binet *et al.*, 2004; Goudet *et al.*, 2004). Identification of the endogenous ligands or even the first tool compounds would be very valuable for the characterization of physiological functions, cellular localizations and therapeutic potential.

In conclusion, the accumulated mutagenesis data for AMs on class C GPCRs almost exclusively maps to one common inter-helical allosteric site for both PAMs and NAMs, which corresponds very well with the allosteric site in the mGlu crystal structures. The combined mGlu-NAM structure complexes, mutagenesis data, sequence comparisons and known NAMs/PAMs lays a stronger foundation for rational design of novel modulators through the construction of high quality homology models. Specifically, structures, mutagenesis data and sequence comparisons have been combined in GPCRdb to create an online tool to design new mutagenesis experiments (Munk *et al.*, 2016). This design aids researchers by selecting the mutations with the highest observed involvement in modulator binding, while avoiding indirect effects through adjacent residues or due to perturbation of the backbone structure. Given the disease relevance of several class C GPCRs, it will be intriguing to explore the integration of data for the identification of potent target- and maybe pathway-specific novel modulators towards more efficient drugs with less adverse effects.

## Supplementary Material

Supplementary Data
