# Supplementary material for: Structural insight to mutation effects uncover a common allosteric site in class C GPCRs
Source: Bioinformatics. 2016 Dec 22;33(8):1116–20. doi: 10.1093/bioinformatics/btw784 (PMC5408886; doi:10.1093/bioinformatics/btw784)
Supplement: Supplementary Data [file btw784_supp.pdf]

Supplementary Information

Structural insight to mutation effects uncover a common allosteric site in class C GPCRs

Kasper Harpsøe, Michael W. Boesgaard, Christian Munk, Hans Bräuner-Osborne and David E. Gloriam

Supplementary Table S1. Overview of physiological functions and disease association of the class C GPCRs

Receptor families/groups	Key physiological functions	Disease association
Group I mGlu receptors ($mGlu_1$, $mGlu_5$)	Potentiation of neuronal excitation throughout the CNS and involved in learning and memory (Niswender and Conn, 2010).	Drug addiction (Pomierny-Chamiolo, et al., 2014), neuroprotection (Caraci, et al., 2012), pain (Montana and Gereau, 2011), Parkinson's disease (Amalric, 2015) and schizophrenia (Vinson and Conn, 2012).
Group II mGlu receptors ($mGlu_2$, $mGlu_3$)	Inhibition of neuronal excitation throughout the CNS (Niswender and Conn, 2010).	Anxiety (Pitsikas, 2014), drug addiction (Pomierny-Chamiolo, et al., 2014), epilepsy (Alexander and Godwin, 2006), neuroprotection (Caraci, et al., 2012), pain (Montana and Gereau, 2011), Parkinson's disease (Amalric, 2015) and schizophrenia (Vinson and Conn, 2012).
Group III mGlu receptors ($mGlu_4$, $mGlu_{6-8}$)	Auto-inhibitory feedback of glutaminergic transmission (Niswender and Conn, 2010).	Anxiety (Pitsikas, 2014), drug addiction (Pomierny-Chamiolo, et al., 2014), pain (Montana and Gereau, 2011) and Parkinson's disease (Amalric, 2015).
$GABA_B$ ($GABA_{B1+B2}$)	Inhibition of neuronal transmission throughout the CNS (Blein, et al., 2000).	Alzheimer's disease, (Chumakov, et al., 2015) Anxiety (Cryan and Kaupmann, 2005), depression (Cryan and Slattery, 2010), drug addiction (Filip, et al., 2015) and absence epilepsy (Onat, et al., 2013).
CaS	Involved in calcium homeostasis, parathyroid hormone release and kidney reabsorption (Ward, et al., 2012).	Genetic disorders involving calcium metabolism (Ward, et al., 2012), Alzheimer's disease (Ward, et al., 2012), Asthma (Yarova, et al., 2015) and colorectal cancer (Aggarwal, et al., 2015).
$GPRC_6$	Generally unknown, putative involvement in bone and energy metabolism, and male fertility (Pi, et al., 2008).	Unknown
TAS1R1-3 (TAS1R1+R3, TAS1R2+R3)	Umami and sweet taste sensing (Li, et al., 2002). Broad expression pattern indicate other physiological functions (Foster, et al., 2013).	Modulation of taste perception and potential in regulating food intake (Servant, et al., 2010).
Orphans (GPR156, GPR158, GPR179 and GPRC5A-D)	Unknown	Unknown

Receptor	AM site	TM
mGlu$_4$	79	72
mGlu$_8$	79	71
mGlu$_7$	79	70
mGlu$_6$	79	67
mGlu$_2$	75	*71*
mGlu$_3$	75	**68**
GPRC5B	64	*49*
GPRC5C	58	*41*
GPRC5A	54	*40*
CaS	50	53
GPRC5D	50	*42*
GABA$_{B1}$	**43**	**41**
GPRC$_6$	39	*48*
TAS1R3	**36**	*35*
GABA$_{B2}$	32	**43**
GPR158	**32**	*34*
TAS1R1	**29**	*46*
GPR156	*29*	*40*
GPR179	**29**	**31**
TAS1R2	**21**	*42*
	52	50

Supplementary Table S2. Class C receptor sequence similarity within the common allosteric site and transmembrane domain, respectively, to the most similar crystallized receptor; mGlu$_1$ - *italic*, mGlu$_5$ - normal and both - **bold**. Receptors above the horizontal bar have a higher similarity within the common allosteric site than the overall transmembrane domain.

Position	Experiments performed	With effect	No. of receptors	With effect	No. of ligands	With effect
1x42	6	2	1	1	3	1
1x46	16	2	2	2	12	1
2x52	8	1	3	1	8	1
2x53	16	2	3	2	8	2
2x56	13	8	3	2	8	4
3x32	30	7	5	4	24	5
3x33	46	7	6	4	41	7
3x36	52	13	5	4	41	11
3x37	13	12	2	2	11	10
3x40	71	44	6	3	44	29
3x41	25	1	5	1	21	1
3x43	31	5	1	1	18	4
3x44	46	26	4	1	30	21
4x42	17	4	4	1	16	4
4x50	7	2	2	1	4	1
4x51	6	1	1	1	6	1
45x49	7	3	2	1	5	2
45x51	14	2	4	1	9	1
45x52	23	4	4	2	19	3
45x53	5	1	2	1	4	1
45x54	4	3	1	1	3	2
5x37	12	1	2	1	10	1
5x40	38	10	4	2	26	3
5x43	64	16	6	5	40	10
5x44	62	23	4	3	37	15
5x47	60	29	5	4	45	22
5x48	32	2	3	1	30	2
6x46	31	20	4	1	26	18
6x50	76	46	6	6	54	37
6x53	59	33	8	6	43	24
6x54	36	13	4	2	24	8
6x57	61	19	6	4	42	15
6x58	24	2	1	1	17	2
7x29	14	7	2	1	2	2
7x33	40	28	5	5	25	15
7x37	19	11	4	4	15	9
7x38	13	1	1	1	11	1
7x40	62	31	5	4	32	18
7x41	55	40	4	1	29	21
7x43	6	2	1	1	3	1
7x47	25	1	2	1	18	1

Supplementary Table S3. Statistics on mutations in the positions showed in the manuscript Figure 2 and 3 showing the number of individual experiments, how many receptors have been examined and how many ligands the mutant have been tested on. Additionally, in each of the three categories the number of ligands affected by mutation in the given position is stated.

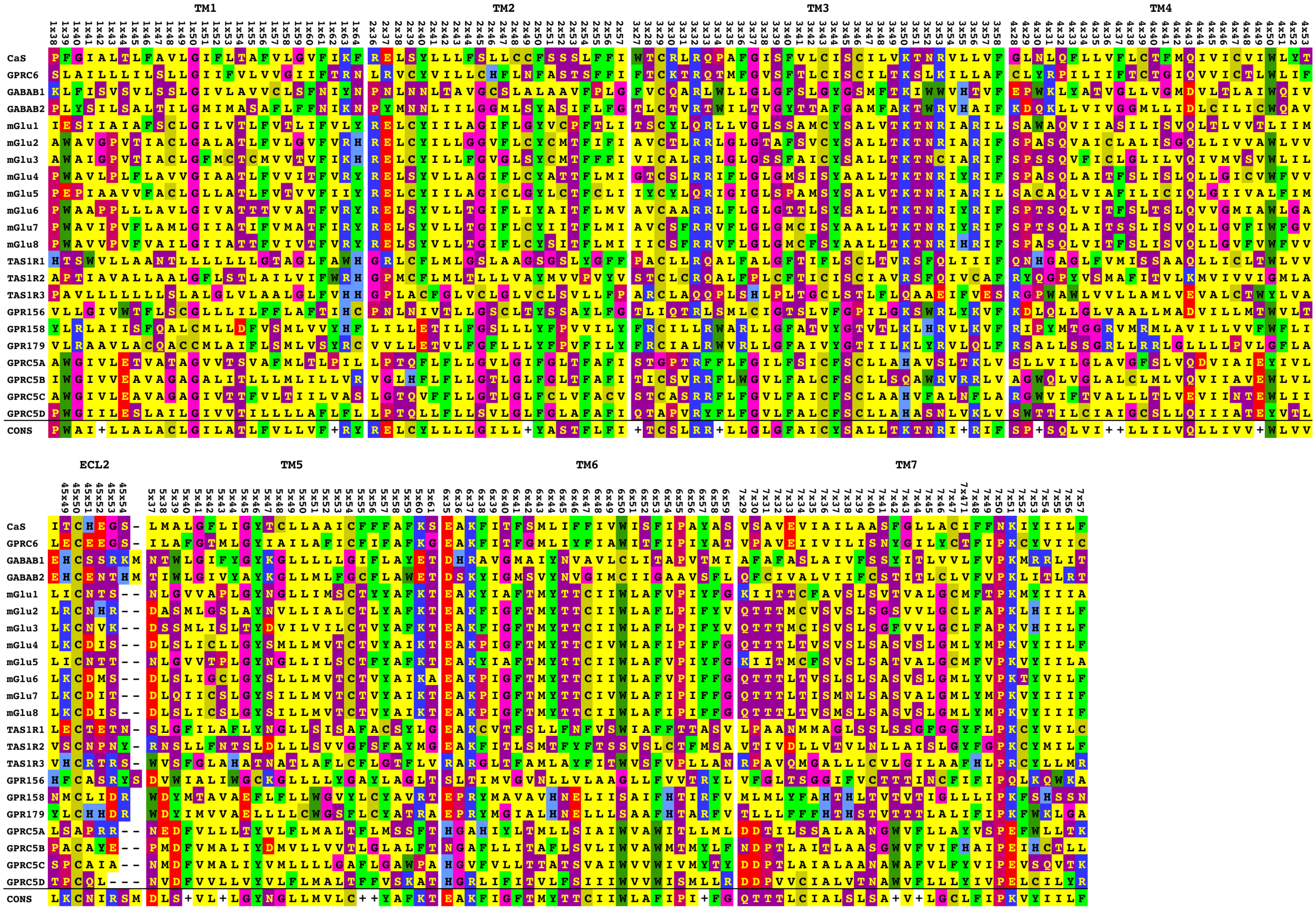

Supplementary Figure S1. Sequence alignment of the 7TM helices and part of the second extracellular loop from the 22 human class C GPCRs.

	2x52	2x53	2x56	3x32	3x33	3x36	3x37	3x40	3x44	4x43	45x49	45x52	5x40	5x43	5x44	5x47	5x51	6x46	6x49	6x50	6x53	6x57	7x29	7x30	7x33	7x36	7x37	7x40
CaS	S	S	F	R	Q	F	G	F	I	Q	T	E	L	L	I	T	A	F	V	W	F	Y	V	S	E	A	I	A
GPRC6	S	T	F	R	Q	F	G	F	I	Q	E	E	F	M	L	I	A	Y	A	W	F	Y	V	P	E	V	I	S
GABAB1	A	V	L	R	L	L	G	F	Y	D	H	S	L	F	Y	K	L	V	L	C	T	T	A	F	A	A	I	S
GABAB2	S	I	F	R	T	L	T	Y	F	D	H	N	L	V	Y	K	M	V	M	C	G	S	Q	F	V	V	I	C
mGlu1	C	P	L	Q	R	V	G	S	Y	Q	I	T	V	P	L	N	I	T	I	W	F	Y	K	I	T	A	V	S
mGlu2	M	T	F	R	R	L	G	F	Y	Q	R	H	M	S	L	N	I	T	I	W	F	F	Q	T	M	S	V	S
mGlu3	M	T	F	R	R	L	G	F	Y	Q	K	V	M	S	L	D	V	T	I	W	F	F	Q	T	M	S	V	S
mGlu4	T	T	M	R	R	L	G	M	Y	Q	K	I	L	L	L	S	M	T	V	W	F	F	Q	T	L	S	V	S
mGlu5	C	T	L	Q	R	I	G	P	Y	Q	I	T	V	P	L	N	I	T	I	W	F	Y	K	I	M	S	V	S
mGlu6	I	T	M	R	R	L	G	T	Y	Q	K	M	L	C	L	S	M	T	I	W	F	F	Q	T	L	S	L	S
mGlu7	I	T	M	R	R	L	G	M	Y	Q	K	I	I	S	L	S	M	T	V	W	F	F	Q	T	L	S	M	S
mGlu8	I	T	M	R	R	L	G	M	Y	Q	K	I	L	S	L	S	M	T	I	W	F	F	Q	T	L	S	M	S
TAS1R1	S	L	F	R	Q	F	A	F	L	Q	E	E	F	A	F	N	S	N	S	W	F	A	L	P	N	A	G	S
TAS1R2	V	V	Y	R	Q	F	P	F	I	K	S	P	L	N	T	D	S	Y	S	S	L	M	V	T	D	V	T	N
TAS1R3	S	V	F	Q	Q	S	H	L	L	E	H	T	F	A	H	N	A	Y	T	W	F	L	R	P	Q	A	L	C
GPR156	S	A	F	R	L	L	C	T	F	D	F	S	I	I	W	K	L	V	A	G	F	R	V	F	T	G	I	C
GPR158	P	V	L	L	R	R	L	F	Y	V	M	I	M	V	A	L	W	E	I	S	F	R	M	L	Y	H	T	T
GPR179	P	V	L	L	R	R	L	F	Y	L	L	H	I	V	A	L	W	E	L	S	F	R	T	L	F	H	T	T
GPRC5A	L	T	F	R	F	F	G	F	F	Q	S	R	F	L	L	V	M	S	I	W	W	L	D	D	L	A	L	N
GPRC5B	L	T	F	R	R	W	G	F	F	Q	A	Y	F	A	L	D	L	S	I	W	W	Y	N	D	L	T	L	S
GPRC5C	L	V	C	R	R	F	G	F	F	E	P	I	F	A	L	V	L	S	I	W	W	Y	D	D	L	A	L	N
GPRC5D	L	A	F	R	Y	F	G	F	F	Q	P	L	F	L	L	V	M	S	I	W	W	L	D	D	V	A	L	N
CONSENSUS	S	T	F	R	R	L	G	F	Y	Q	K	I	+	+	L	N	M	T	I	W	F	+	Q	T	L	A	L	S
AROMATIC	0	0	55	0	9	36	5	64	77	0	18	14	32	5	23	0	9	18	0	73	86	55	0	14	9	9	0	0
POLAR	32	50	0	91	82	14	9	14	0	91	64	55	0	23	9	73	9	68	14	14	5	23	68	55	32	45	14	82
PHOB/ALI	68	50	45	9	9	50	23	23	23	9	32	41	68	73	73	27	82	14	86	9	5	23	32	32	59	50	82	18
Gly	0	0	0	0	0	0	68	0	0	0	0	0	0	0	0	0	0	0	0	5	5	0	0	0	0	5	5	0

Supplementary Figure S2. Sequence alignment of the 28 positions involved in the common allosteric binding site in the 22 human class C GPCRs. Below is the consensus sequence and percentage conservation of the position as aromatic, polar, hydrophobic/aliphatic and glycine.

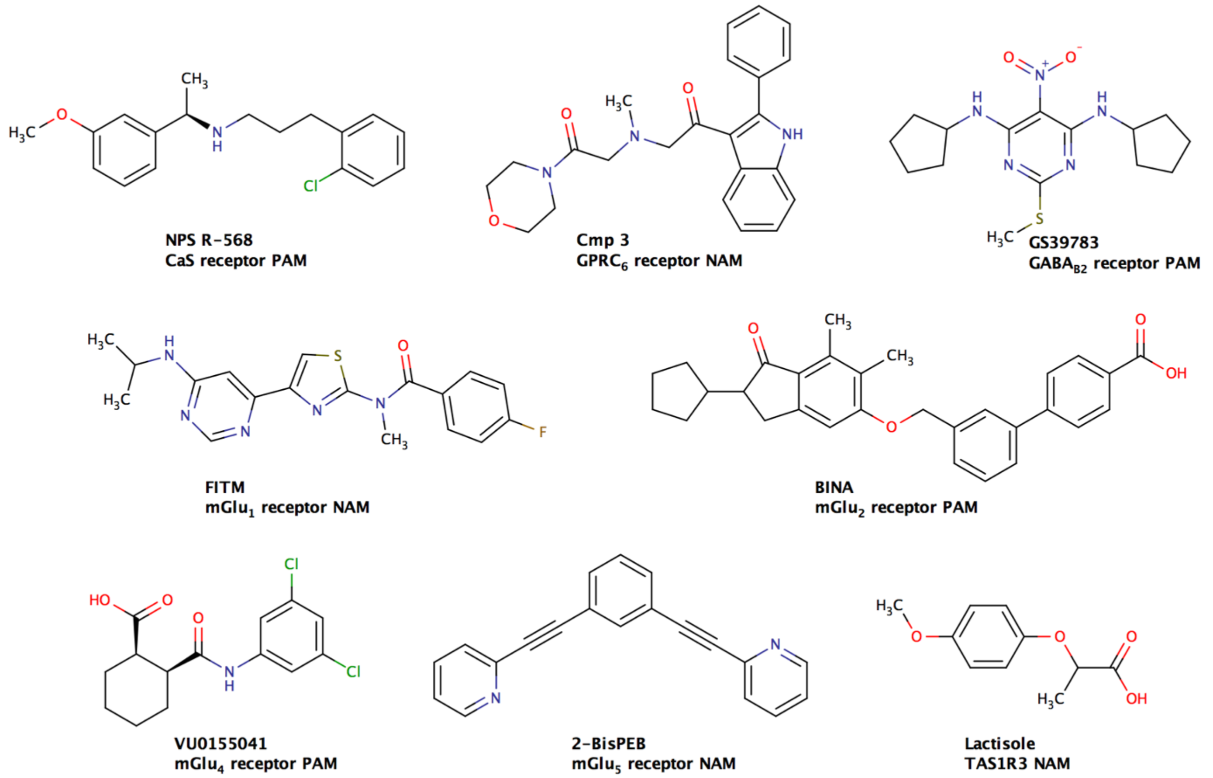

Supplementary Figure S3. Representative allosteric modulators from the class C GPCR mutagenesis experiments (Supplementary Table 1). The modulators are structurally diverse, showing that different ligand chemotypes can be accommodated in the overlapping binding sites. This is consistent with the sequence diversity within the allosteric binding site positions (Supplementary Figure 2). Chemical structures were prepared with MarvinSketch 6.2.1, 2014, ChemAxon

Supplementary References

Aggarwal, A., *et al.* (2015) The calcium-sensing receptor: A promising target for prevention of colorectal cancer. *Biochim. Biophys. Acta*, **1853**, 2158-2167.

Alexander, G.M. and Godwin, D.W. (2006) Metabotropic glutamate receptors as a strategic target for the treatment of epilepsy. *Epilepsy. Res.*, **71**, 1-22.

Amalric, M. (2015) Targeting metabotropic glutamate receptors (mGluRs) in Parkinson's disease. *Curr. Opin. Pharmacol.*, **20**, 29-34.

Blein, S., *et al.* (2000) The metabotropic GABA receptor: molecular insights and their functional consequences. *Cell. Mol. Life Sci.*, **57**, 635-650.

Caraci, F., *et al.* (2012) Metabotropic glutamate receptors in neurodegeneration/neuroprotection: still a hot topic? *Neurochem. Int.*, **61**, 559-565.

Chumakov, I., *et al.* (2015) Combining two repurposed drugs as a promising approach for Alzheimer's disease therapy. *Sci. Rep.*, **5**, 7608.

Cryan, J.F. and Kaupmann, K. (2005) Don't worry 'B' happy!: a role for GABA(B) receptors in anxiety and depression. *Trends Pharmacol. Sci.*, **26**, 36-43.

Cryan, J.F. and Slattery, D.A. (2010) GABAB receptors and depression. Current status. *Adv. Pharmacol.*, **58**, 427-451.

Filip, M., *et al.* (2015) GABAB receptors as a therapeutic strategy in substance use disorders: focus on positive allosteric modulators. *Neuropharmacology*, **88**, 36-47.

Foster, S.R., *et al.* (2013) Expression, regulation and putative nutrient-sensing function of taste GPCRs in the heart. *PLoS One*, **8**, e64579.

Li, X., *et al.* (2002) Human receptors for sweet and umami taste. *Proc Natl Acad Sci U S A*, **99**, 4692-4696.

Montana, M.C. and Gereau, R.W. (2011) Metabotropic glutamate receptors as targets for analgesia: antagonism, activation, and allosteric modulation. *Curr. Pharm. Biotechnol.*, **12**, 1681-1688.

Niswender, C.M. and Conn, P.J. (2010) Metabotropic glutamate receptors: physiology, pharmacology, and disease. *Annu. Rev. Pharmacol. Toxicol.*, **50**, 295-322.

Onat, F.Y., *et al.* (2013) The involvement of limbic structures in typical and atypical absence epilepsy. *Epilepsy Res.*, **103**, 111-123.

Pi, M., *et al.* (2008) GPRC6A null mice exhibit osteopenia, feminization and metabolic syndrome. *PLoS One*, **3**, e3858.

Pitsikas, N. (2014) The metabotropic glutamate receptors: potential drug targets for the treatment of anxiety disorders? *Eur. J. Pharmacol.*, **723**, 181-184.

Pomierny-Chamiolo, L., *et al.* (2014) Metabotropic glutamatergic receptors and their ligands in drug addiction. *Pharmacol. Ther.*, **142**, 281-305.

Servant, G., *et al.* (2010) Positive allosteric modulators of the human sweet taste receptor enhance sweet taste. *Proc. Natl. Acad. Sci. USA*, **107**, 4746-4751.

Vinson, P.N. and Conn, P.J. (2012) Metabotropic glutamate receptors as therapeutic targets for schizophrenia. *Neuropharmacology*, **62**, 1461-1472.

Ward, B.K., *et al.* (2012) The role of the calcium-sensing receptor in human disease. *Clin. Biochem.*, **45**, 943-953.

Yarova, P.L., *et al.* (2015) Calcium-sensing receptor antagonists abrogate airway hyperresponsiveness and inflammation in allergic asthma. *Sci. Transl. Med.*, **7**, 284ra260.
